# Chewing the Fat: The Conserved Ability of DNA Viruses to Hijack Cellular Lipid Metabolism

**DOI:** 10.3390/v11020119

**Published:** 2019-01-29

**Authors:** Philip T. Lange, Michael Lagunoff, Vera L. Tarakanova

**Affiliations:** 1 Department of Microbiology and Immunology, Medical College of Wisconsin, Milwaukee, WI 53226, USA; plange@mcw.edu; 2 Department of Microbiology, University of Washington, Seattle, WA 98101, USA; lagunoff@uw.edu; 3 Molecular and Cellular Biology Program, University of Washington, Seattle, WA 98101, USA; 4 Cancer Center, Medical College of Wisconsin, Milwaukee, WI 53226, USA

**Keywords:** DNA virus, lipids, fatty acids, cholesterol, herpesvirus, MHV68

## Abstract

Viruses manipulate numerous host factors and cellular pathways to facilitate the replication of viral genomes and the production of infectious progeny. One way in which viruses interact with cells is through the utilization and exploitation of the host lipid metabolism. While it is likely that most—if not all—viruses require lipids or intermediates of lipid synthesis to replicate, many viruses also actively induce lipid metabolic pathways to sustain a favorable replication environment. From the formation of membranous replication compartments, to the generation of ATP or protein modifications, viruses exhibit differing requirements for host lipids. Thus, while the exploitation of lipid metabolism is a common replication strategy, diverse viruses employ a plethora of mechanisms to co-opt these critical cellular pathways. Here, we review recent literature regarding the exploitation of host lipids and lipid metabolism specifically by DNA viruses. Importantly, furthering the understanding of the viral requirements for host lipids may offer new targets for antiviral therapeutics and provide opportunities to repurpose the numerous FDA-approved compounds targeting lipid metabolic pathways as antiviral agents.

## 1. Overview of Lipid Metabolism

Cellular metabolism encompasses numerous pathways that mediate the synthesis and degradation of energy-providing compounds and molecular building blocks. Importantly, thanks to a number of landmark studies and the seemingly universal importance of the metabolism for diverse viruses, the topic of cellular metabolism is enjoying renewed appreciation amongst virologists. In this review, we will discuss the interplay between cellular lipid metabolism and DNA virus infection, with a particular focus on the synthetic pathways.

Lipid metabolism comprises multiple cellular pathways that mediate the synthesis and breakdown of compounds such as fatty acids, sterols, and other molecules. Similar to other metabolic pathways, many of the main players have been elucidated; however, the regulatory complexity of lipid metabolism is still an active area of investigation.

## 2. Transcriptional Regulation of Lipid Metabolism

The transcriptional regulation of lipogenesis occurs through the activation of several key transcription factors. Perhaps most notable of these are the sterol regulatory element-binding proteins (SREBPs) of which there are three proteins that are transcribed from two genes. The isoforms SREBP-1a and SREBP-1c are splice variants of a single gene, whereas SREBP2 is encoded at a distinct locus. Canonically, SREBP-1a acts as an activator of all sterol response element (SRE)-containing promoters, whereas SREBP-1c and SREBP-2 induce the expression of genes involved in fatty acid synthesis and cholesterol synthesis, respectively (reviewed in [1,2]).

All newly-synthesized SREBPs are complexed in the ER membrane with the SREBP cleavage activation protein (SCAP). This complex further associates with the sterol-sensitive insulin-induced genes (Insig-1 and Insig-2), which are responsible for sequestering the SCAP–SREBP complex in the ER. Cholesterol directly interacts with SCAP, stabilizing the association between the SCAP–SREBP complex and Insigs. Similarly, several oxysterols, oxidized derivatives of cholesterol, enhance the sequestration of SCAP–SREBP complexes through interactions with Insigs. When cellular sterol levels are low, the SCAP–SREBP complex dissociates from the Insig and traffics to the Golgi apparatus, where SREBPs are cleaved by Site-1 protease and Site-2 protease, releasing the N-terminal portion of the SREBP. This mature SREBP (also called nuclear SREBP, or nSREBP) translocates to the nucleus to mediate the transcription of genes with SRE-containing promoters.

Importantly, while SREBPs regulate the expression of many enzymes involved in the synthesis of lipids, they are not the only means of regulation. Liver X receptors (LXRs) represent another important axis of lipid homeostasis [3]. LXRs are ligand-activated transcription factors of the nuclear receptor superfamily. There are two LXR isoforms (LXRα and LXRβ) that are transcribed from distinct genes. These two isoforms share significant sequence homology, but differ in their cellular expression profiles. Generally, LXRα is highly expressed in metabolically active cell types, such as hepatocytes, adipocytes, and myocytes [4,5,6]. Interestingly, LXRα expression is also high in macrophages and other select subsets of hematopoietic cells. LXRβ, on the other hand, is ubiquitously expressed. Recognition of liver X response elements (LXREs) requires heterodimerization between LXR and another nuclear receptor, retinoid X receptor (RXR) [4,5]. Ligand-mediated activation, physiologically by oxysterols and desmosterol, or non-physiologically by artificial synthetic ligands, leads to the induction of genes generally involved in fatty acid synthesis and cholesterol efflux [7]. Furthermore, as LXRs heterodimerize with RXRs, RXR ligands are also capable of inducing the expression of LXRE-containing genes, and the presence of both LXR and RXR ligands can have an additive or synergistic effect on gene expression [5,8]. Importantly, expression of the aforementioned transcription factor SREBP-1c is promoted by LXR [9].

## 3. Enzymatic Regulation of Lipid Metabolism

**Figure 1:**
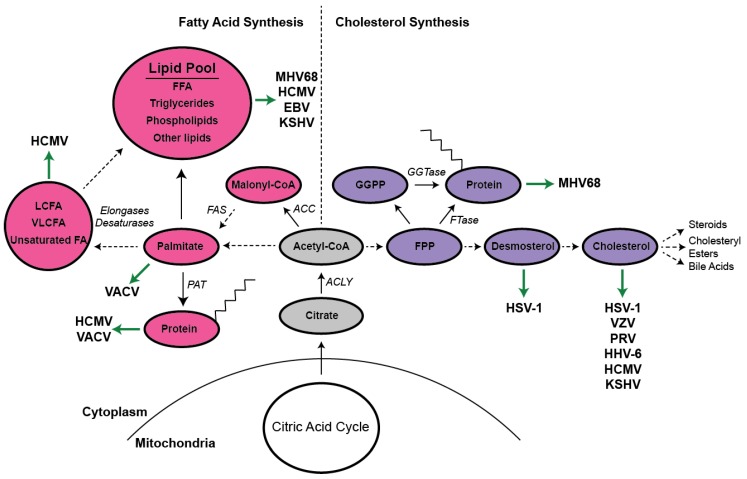
Summary of the interplay between DNA virus infection and cellular lipid metabolism. This model illustrates a number of enzymes and intermediates involved in the synthesis of cholesterol (purple, right side), fatty acids (pink, left side), and related compounds. The precursor acetyl-CoA feeds both the mevalonate and fatty acid synthesis pathways. Virus interactions with these compounds are indicated by green arrows. Solid black arrows indicate the direct conversion of one molecule to the next, whereas dashed arrows indicate additional steps that, for the sake of simplicity, are not depicted. To conserve space, prenyl and palmitoyl moieties are depicted with only eight carbons. MHV68 (murine herpesvirus 68), HCMV (human cytomegalovirus), EBV (Epstein–Barr virus), KSHV (Kaposi’s sarcoma herpesvirus), VACV (vaccinia virus), HSV1 (herpes simplex virus-1), ACC (acetyl-CoA carboxylase), FAS (fatty acid synthase), PAT (palmitoyl acyl transferase), ACLY (ATP citrate lyase), LCFA (long chain fatty acids), VLCFA (very long chain fatty acids), FA (fatty acids), FFA (free fatty acids), FPP (farnesyl pyrophosphate), GGPP (geranylgeranyl pyrophosphate), FTase (farnesyl transferase), GGTase (geranylgeranyl transferase).

As previously mentioned, SREBP-1c and SREBP2 generally induce the transcription of genes involved in fatty acid synthesis and cholesterol synthesis, respectively [1,2]. Each of these pathways utilizes the common precursor moiety, acetyl-CoA. In the synthesis of fatty acids (Figure 1), acetyl-CoA carboxylase (ACC) converts acetyl-CoA to malonyl-CoA through the addition of CO_2_. This is the rate-limiting step of fatty acid synthesis, and as such, ACC is subjected to multiple mechanisms of regulation. The multi-functional enzyme complex, fatty acid synthase (FAS), then initiates the subsequent steps of fatty acid synthesis, first joining acetyl-CoA and malonyl-CoA molecules to create butyryl-CoA. This immature fatty acid is further lengthened through the addition of two carbon units derived from additional malonyl-CoA moieties, until the 16-carbon fatty acid, palmitate, is generated. A cadre of desaturases, elongases, and other enzymes can further process palmitate, ultimately allowing for the formation of hundreds of species of fatty acids, phospholipids, triglycerides, and other lipids [10]. Importantly, in a converse process, fatty acids can be degraded though a process known as β-oxidation to generate acetyl-CoA for oxidation in the citric acid cycle.

Cholesterol synthesis similarly begins from the precursor acetyl-CoA; however, the enzymes and processes involved in cholesterol synthesis are distinct (Figure 1). The rate-limiting step of cholesterol biosynthesis, generally referred to as the mevalonate pathway, is catalyzed by 3-hydroxy-3-methylglutaryl-CoA reductase (HMGCR). Like ACC, HMGCR is subjected to numerous forms of regulation, including sterol-mediated transcriptional repression and proteasomal degradation. Additionally, a therapeutically important class of small molecule inhibitors, collectively known as “statins,” competitively bind the active site of HMGCR, inhibiting the synthesis of mevalonate and, subsequently, cholesterol. These drugs are widely prescribed for the treatment of hypercholesterolemia and, notably, are actively being investigated as potential antiviral agents [11].

In addition to cholesterol, the synthesis of many other important molecules, such as ubiquinone, steroids, cholesteryl esters, bile acids, heme A, and numerous vitamins and isoprenoids, requires the mevalonate pathway. The isoprenoid farnesyl-pyrophosphate (FPP) represents an interesting branch point in the pathway, as it can either be converted to squalene, and ultimately cholesterol, utilized for ubiquinone or heme synthesis, or covalently attached to proteins through a process called prenylation [12,13]. Another prenyl moiety, geranylgeranyl pyrophosphate (GGPP), is synthesized from FPP and isopentenyl pyrophosphate. Protein prenylation with FPP, a hydrophobic 15-carbon unit, is mediated by the enzyme farnesyl transferase. Similarly, the attachment of GGPP, a 20-carbon molecule, is carried out by geranylgeranyl transferase. These enzymes recognize proteins with a CAAX motif, where “C” is the cysteine that is prenylated, “A” is any aliphatic amino acid, and “X” can be one of several amino acids and determines the specificity for farnesylation or geranylgeranylation. This modification facilitates numerous signaling pathways, most notably small GTPase signaling, by altering protein localization and protein–protein interactions [13].

Importantly, it has become increasingly clear that cellular lipid metabolism facilitates the replication of diverse viral families, and many viruses encode genes that regulate the activity of lipogenesis. Despite the remarkable diversity amongst human viral pathogens, both RNA and DNA viruses have been shown to co-opt cellular lipids to facilitate similar processes. For a review on RNA virus infection and host lipid metabolism, please see the counterpart to this review by Dr. Perera (cite Rushika’s RNA virus review here). This review will focus on the current understanding of the interactions between DNA viruses and lipid metabolism.

## 4. Herpesviruses

There are numerous families of DNA viruses that represent significant burdens to human health. With respect to host lipid metabolism, most of the published studies have examined the role that these pathways play during herpesvirus infection. Herpesviridae is a family of ancient double-stranded DNA viruses that have co-evolved with their hosts over millions of years. Herpesviruses, with the exception of most alphaherpesviruses, display significant species-specificity, likely due, at least in part, to their co-evolution with animal hosts. Currently, there are three subfamilies under the Herpesviridae umbrella: alphaherpesvirinae, betaherpesvirinae, and gammaherpesvirinae. While these viral subfamilies are generally quite distinct, they exhibit a number of important shared features. A unique characteristic of herpesviruses is their ability to undergo two distinct life cycles: lytic replication and latency. The former is defined by the production of infectious progeny virions, whereas latency is primarily a quiescent state in which the viral genome is maintained as a circular episome within the nucleus of an infected cell and exhibits minimal viral gene expression. Generally, the lytic replication of herpesviruses utilizes many viral replication factors, including a viral DNA polymerase. In contrast, viral latency is maintained in the absence of expression of most viral genes, and the viral genome is copied once per cell division by the host DNA polymerase. Numerous thorough reviews have been written on the two herpesvirus life cycles [14,15,16,17,18]. Importantly, due to the drastic differences between lytic replication and latency, it is likely that each life cycle has different effects on, and requirements for, host-derived biosynthetic compounds. Notably, within the herpesvirus arena, there is growing interest to understand the interplay between viral infection and host lipid metabolism.

### 4.1. Alphaherpesviruses

The alphaherpesviruses comprise three known human pathogens: herpes simplex virus-1 (HSV-1), HSV-2, and varicella zoster virus (VZV). Additionally, pseudorabies virus (PRV), an endemic porcine pathogen, is widely employed as a model system of alphaherpesvirus biology. While, as mentioned above, the alphaherpesviruses generally exhibit broader host range than their beta- and gammaherpesvirus counterparts, they are defined, in part, by their tropism for sensory ganglia, as well as their genome sequence and replication time [19]. As they generally exhibit neurotropism, the alphaherpesviruses present with unique pathologies. HSV-1 and HSV-2 cause cold sores, genital lesions, and, rarely, meningitis and encephalitis. VZV is the causative agent of chickenpox and shingles.

Initial reports of how alphaherpesvirus infection modulates host lipid metabolism were published in the mid 1980s. Jerkofsky et al. discovered that the VZV infection of MRC-5 cells resulted in profound changes in neutral lipid synthesis [20]. This study examined 11 different VZV isolates, and noted that every isolate induced a shift toward neutral lipid synthesis with respect to the synthesis of total lipids. However, when relative rates of total neutral lipid and triglyceride synthesis were determined, it was unveiled that several of the strains dramatically reduced the synthesis rate of these compounds, while others significantly increased the rate of synthesis. These seemingly confounding results suggest that different VZV strains may have differing requirements for host lipids. Alternatively, as interferon (IFN) signaling and cellular lipid metabolism are intimately linked, this finding may represent the differential induction of host antiviral responses by each of the VZV isolates.

Other early investigations into the interactions between alphaherpesviruses and lipid metabolism identified HSV-1-dependent changes in the synthesis of the trafficking and signaling lipids, phosphoinositides [21]. Specifically, it was determined that the relative rates of phosphatidylinositol 4,5-bisphosphate (PIP_2_) and phosphatidylinositol 4-phosphate (PIP) synthesis are increased and decreased, respectively, by the early genes of HSV-1 [21,22]. Thus, HSV-1 likely interferes with host lipid metabolism to affect cellular signaling pathways, including the intracellular trafficking of host and/or viral proteins.

Notably, while these observations regarding VZV and HSV-1 infections were first reported over three decades ago, few to no related reports regarding alphaherpesviruses and lipids were published until relatively recently. It is now appreciated that, similar to numerous RNA viruses, the efficient entry of HSV-1, VZV, and PRV requires cellular membrane cholesterol [23,24,25,26,27]. Additionally, the presence of the cholesterol precursor, desmosterol, appears to partially rescue HSV-1 entry in the absence of cellular cholesterol [24]. Interestingly, in contrast to other reports [23,24], Gianni et al. provide evidence that cellular membrane cholesterol is critical for HSV-1 entry only in the presence of α_V_β_3_-integrins [27]. Further, this study demonstrates that α_V_β_3_-integrin expression dictates the pathway by which HSV-1 enters. It is worth noting, however, that the cholesterol depletion methods differed between the aforementioned studies, potentially confounding direct comparison of the results.

In addition to the clear importance of cellular cholesterol in the entry processes of alphaherpesviruses, there is also evidence that cholesterol facilitates HSV-1 replication post-entry [28]. Specifically, Wudiri and Nicola observed that the depletion of cellular cholesterol up to nine hours post-infection significantly abrogates HSV-1 replication [28]. Fascinatingly, cells lacking the ability to convert desmosterol to cholesterol are capable of supporting HSV-1 infection; however, viral replication is modestly attenuated [28]. As herpesvirus membranes are derived from host cell organelle membranes, one would expect the lipid composition of the viral envelope to regulate viral entry and/or egress. As expected, viral membrane cholesterol has indeed been demonstrated to facilitate HSV-1 entry [29], adding further emphasis to the importance of sterols during the alphaherpesvirus infection cycle. Finally, following the infection of cells that are unable to convert desmosterol to cholesterol, the resulting, presumably desmosterol-containing, virions exhibited the same specific infectivity as cholesterol-containing HSV-1 [28].

Sterols, of course, represent an important component of host and viral membranes; however, the major constituents of these biological structures are phospholipids. As herpesviruses follow a complex egress pathway, first budding into, then out of the perinuclear space before becoming re-enveloped at the trans-Golgi network, it is conceivable that herpesvirus infection may also perturb phospholipid metabolism. The first attempt to measure phospholipid synthesis during HSV-1 infection was reported by Asher et al. via the incorporation of [^3^H]choline [30]. Interestingly, while the authors were able to detect the incorporation of [^3^H] choline-containing lipids in newly synthesized viral particles, they did not observe any change in cellular phospholipid synthesis. More recent reports, however, have observed increased de novo phospholipid synthesis upon HSV-1 infection [31,32]. Thus, the extent to which alphaherpesviruses manipulate cellular phospholipid metabolism remains largely undetermined.

### 4.2. Betaherpesviruses

There are four betaherpesviruses known to infect humans: human cytomegalovirus (HCMV), and human herpesviruses 6A, 6B, and 7 (HHV-6A, HHV-6B, HHV-7). Among the betaherpesviruses, HCMV, the leading infectious cause of congenital defects [33], is also the most heavily studied with respect to interaction with the host lipid metabolism. HCMV, depending on the strain (lab-adapted vs. clinical isolates), is able to infect and replicate in several cell types in vitro, including epithelial cells, endothelial cells, and fibroblasts. Additionally, HCMV infection of myeloid cells is believed to be critical for the dissemination of the virus in vivo [14], and viral latency is maintained in CD14^+^ mononuclear cells of the peripheral blood, as well as CD33^+^/CD34^+^ hematopoietic progenitor cells in the bone marrow [34,35]. HHV-6A, 6B, and 7, collectively known as the roseoloviruses due to their association with the disease roseola infantum, display varying degrees of selectivity for CD4^+^ T cells.

Like the alphaherpesviruses, HCMV and the roseoloviruses also utilize cholesterol-dependent cell entry processes [36,37,38,39,40], highlighting an evolutionarily conserved feature of herpesviruses. Additionally, the attenuation of cholesterol synthesis via treatment with statins reduced HCMV infection of human umbilical vein endothelial cells [41]. The antiviral mechanism of statin treatment in this system was not determined; however, it seems likely that it may involve the attenuation of cholesterol-dependent entry. Like HSV-1, HCMV membrane cholesterol also contributes to the infectivity of virions in cell culture [42]. Further, the HCMV glycoprotein B (gB) has been reported to be modified by palmitoylation [43], a protein modification by the fatty acid palmitate. Palmitoylation was found to promote gB association with lipid rafts and support the fusogenic activity of HCMV in epithelial cells [43].

The ability of HCMV to increase glucose uptake and glycolysis has been appreciated for decades [44]; however, the effect of betaherpesvirus infection on lipid metabolism has been neglected until recently. Thanks to renewed interest in the multifaceted roles of metabolism during viral infection, we now know that dramatic lipogenic reprogramming occurs upon HCMV infection [45,46,47,48,49]. A fascinating study by Munger et al. [47] utilized cutting edge metabolic flux profiling to follow the incorporation of ^13^C-labeled nutrients into downstream metabolites. Remarkably, they report that much of the glucose imported during HCMV infection is used to generate citrate in the citric acid cycle, which is then shuttled into the cytoplasm where the two-carbon units generated by ATP citrate lyase are used to support fatty acid synthesis [47]. When measured directly, HCMV-infected fibroblasts showed an approximate 5 to 10-fold increase in the incorporation of glucose-derived carbons into newly synthesized fatty acids compared to uninfected controls [47]. Other reports have confirmed this extraordinary increase in lipid synthesis upon de novo HCMV infection [45,46,48,49], and expanded upon the mechanism of HCMV-induced lipogenesis.

Consistent with the increase in lipogenesis, the expression of the enzyme acetyl-CoA carboxylase (ACC), which catalyzes the rate-limiting step of fatty acid synthesis, is significantly elevated by 48 h post infection with HCMV in an mTOR-dependent manner [48]. The predominant isoform of ACC in most tissues, ACC1, is negatively regulated by phosphorylation at Ser79 [50,51]. Interestingly, although HCMV infection results in increased ACC1 expression and specific activity, Ser79-phosphorylated ACC1 is also elevated [48]. Despite this somewhat confounding increase in phosphorylated ACC1, inhibition of ACC1, both pharmacologically and via siRNA treatment, significantly attenuates the replication of HCMV [48].

Concordant with the increased expression of ACC1, the infection of human foreskin fibroblasts with HCMV leads to elevated expression of other critical lipogenic genes such as fatty acid synthase, ATP citrate lyase, and stearoyl-CoA desaturase 1 [45]. This has been proposed to result from the activation of SREBP-1a and/or SREBP-1c, as depletion of SCAP, a protein required for SREBP activity, significantly attenuates the infection-induced increase in lipogenic gene expression [45]. Additionally, the inhibition of SREBP-1 activation via depletion of SCAP attenuates HCMV replication in fibroblasts [45]. The mechanism of HCMV-induced SREBP-1 activation is currently thought to involve a dramatic increase in the expression of the PRK-like endoplasmic reticulum kinase (PERK), which has previously been demonstrated to facilitate SREBP activation [46,52].

Additional reports suggest that the glucose-sensitive transcription factor, carbohydrate-responsive element binding protein (ChREBP), independent of SREBP-1 activation, contributes to the upregulation of lipogenic genes upon HCMV infection [53,54]. A fascinating mechanism has been proposed for the HCMV-mediated upregulation of ChREBP. Specifically, the HCMV viral mitochondrial inhibitor of apoptosis (vMIA) has been demonstrated to co-opt viperin, a host interferon stimulated gene, and re-localize it to the mitochondria [55]. At the mitochondria, viperin is proposed to attenuate fatty acid β-oxidation, thereby depleting intracellular ATP levels, leading to the activation of AMPK, expression of the glucose transporter GLUT4, and ultimately, activation of ChREBP [53,55]. Thus, while all of the mechanistic processes that underlie HCMV-induced lipogenesis remain to be firmly established, it is clear that HCMV infection usurps host lipogenic pathways for its own benefit. Additionally, despite regulating the expression of both SREBP-1 and ChREBP, the role of LXRs in HCMV-induced lipogenesis has not been examined.

With respect to the mechanisms by which lipogenesis supports HCMV replication, a recent siRNA screen points to a critical role for very long chain fatty acids (VLCFAs, >20 carbons) in the production of progeny HCMV virions [56]. Specifically, the authors of this study report a significant increase in the cellular concentration of fatty acids of 26 carbons or longer, most of which are saturated. Further, many of these VLCFAs were detected in viral membranes and appear to facilitate both the production of progeny virions and the infectivity of said virions. Long chain fatty acids (LCFAs, 14–20 carbons) and VLCFAs can be further lengthened by a group of enzymes known as fatty acid elongases (ELOVLs). Purdy et al. have recently discovered that ELOVL7, an elongase with high activity towards 18-carbon saturated and monounsaturated fatty acyl-CoAs [57,58], facilitates HCMV replication and infectivity [59]. The expression of *ELOVL7* is significantly increased by 24 h post infection, and is >100-fold elevated by 72 h post infection of MRC-5 fibroblasts [59]. The elevated expression of *ELOVL7* transcript and protein following infection was found to partially depend on the expression of the viral protein UL38 [59], which has previously been demonstrated to manipulate cellular metabolism by indirectly increasing mTOR activity [60,61,62,63,64]. Ectopic expression of UL38 was sufficient to elevate ELOVL7 expression; however, it was insufficient to increase fatty acid elongation, possibly due to lack of induction of upstream lipogenic enzymes [59].

In addition to promoting fatty acid synthesis, HCMV has also been demonstrated to modify cholesterol synthesis [65] and trafficking [42,66]. HCMV infection has been proposed to increase cholesterol efflux through two recently reported mechanisms. First, the viral protein US28 was found to enhance cholesterol efflux through actin rearrangements requiring CDC42 [66]. The activity of CDC42 was found to enhance the binding of the extracellular cholesterol acceptor, apolipoprotein A-1 (apoA-1), to the cell membrane. Remarkably, this efflux was independent of ATP binding cassette transporter A1 (ABCA1), the protein responsible for transferring cholesterol to apoA-1, suggesting that HCMV utilizes an alternative mechanism to enhance cholesterol efflux [66]. The benefit that this confers to the virus is unclear; however, the authors propose that the disruption of lipid rafts may attenuate inflammatory signaling pathways.

A separate proteomic analysis identified increased expression of low density lipoprotein receptor-related protein 1 (LRP1) in HCMV-infected fibroblasts [42]. LRP1 is a transmembrane receptor with roles in numerous cellular processes, including lipid metabolism, hemostasis, cell migration, and clearance of apoptotic cells (reviewed in [67,68]). Expression of LRP1 in fibroblasts infected with HCMV was found to negatively correlate with cellular cholesterol content [42]. Additionally, the transient knockdown of LRP1 before infection produced HCMV virions that were subsequently significantly more infectious than virions released from control cells, possibly due to elevated cholesterol content in the viral membranes [42].

### 4.3. Gammaherpesviruses

There are two human gammaherpesviruses: Epstein–Barr virus (EBV) and Kaposi’s sarcoma herpesvirus (KSHV), also known as human herpesvirus-8 (HHV-8). EBV is the etiologic agent of infectious mononucleosis in teens and is also associated with endemic Burkitt lymphoma, nasopharyngeal carcinoma as well as post-transplant and other immunosuppressed B-cell lymphomas. KSHV is the etiologic agent of Kaposi’s sarcoma (KS) as well as two lymphoproliferative diseases, primary effusion lymphoma and the plasmablastic form of multicentric Castleman’s disease. The main tumor cell of KS is the spindle cell, a cell that expresses markers of endothelium. In late-stage tumors, all spindle cells contain KSHV, predominantly in the latent state, although a low percentage of cells express markers of lytic replication. Primary effusion lymphomas and multicentric Castleman’s disease (MCD) are B-cell diseases that also predominantly maintain latent infection with a percentage of cells undergoing lytic infection with MCD having a higher lytic component than the other KSHV associated diseases.

Similar to the alpha and beta herpesviruses, KSHV requires cholesterol for entry. The inhibition of lipid rafts through the sequestration of cholesterol in the rafts by different drugs led to a decrease in KSHV entry into cells [69]. Lipid rafts are also important for KSHV egress. The inhibition of cholesterol in lipid rafts led to a significant decrease in the production of extracellular virus particles without altering the replication of KSHV DNA inside the cells [70].

While cholesterol in lipid rafts is important for the entry and egress of KSHV, fatty acid synthesis is important for virion production [71]. Prevention of glycolysis or glutaminolysis leads to an inhibition of viral gene expression and KSHV DNA replication. However, the inhibition of fatty acid synthesis by the ACC1 inhibitor, TOFA, does not affect KSHV gene expression or viral DNA replication or production. Additionally, there were large numbers of intracellular capsids produced when fatty acid synthesis was inhibited. However, there is a dramatic decrease in the production of extracellular virions despite the production of intracellular virions. Therefore, fatty acid synthesis appears to be required for the proper maturation and egress of KSHV.

Fatty acid synthesis is also critical during KSHV latent phase infection. A metabolomics study of endothelial cells latently infected with KSHV found that over half of the measured long chain fatty acids were increased by KSHV infection [72]. There is a concomitant increase in lipid droplets in endothelial cells latently infected with KSHV. The inhibition of either ACC1 or fatty acid synthase (FASN) leads to significantly increased cell death of latently infected endothelial cells as compared to their mock-infected counterparts. Therefore, fatty acid synthesis is both increased and required during KSHV latent phase infection in endothelial cells. Increased cholesterol esters and triglycerides were also found following latent infection of HUVEC cells [73], although a recent paper showed that KSHV miRNAs decreased cholesterol during infection of HUVECs [74]. Fatty acid synthesis is also important for the survival of primary effusion lymphomas. FASN was found in high levels in primary effusion lymphomas as compared to primary B-cells along with higher levels of lipid droplets [75]. There was no increase in levels of fatty acid oxidation in PEL cells, indicating that the increased lipid droplets came from fatty acid synthesis. As with endothelial cells, the inhibition of FASN with C75 led to increased cell death in the primary effusion lymphoma cells. The inhibition of the increased glycolysis in primary effusion lymphoma cells also inhibited the fatty acid synthesis, indicating the linkage between glycolysis and fatty acid synthesis in these cells. Why increased fatty acid synthesis might be necessary for the survival of latently infected endothelial cells and B-cells is not yet clear, but further studies are ongoing to determine the role of fatty acid synthesis or downstream metabolic processes during latent infection.

In addition to increased fatty acid synthesis, KSHV latent infection leads to increased peroxisome biogenesis [76]. Peroxisomes are small organelles that contribute to the breakdown of very long chain fatty acids. Peroxisomes also contain enzymes important for the relief of oxidative stress. Peroxisomes take up specific long chain fatty acids including 24:6n3 through the ATP binding cassette subfamily D member 3 (ABCD3) transporter and use the enzyme Acyl-CoA oxidase 1 (ACOX1) to convert it to docosahexaenoate. Knockdown of either ABCD3 or ACOX1 led to significantly increased cell death in latently infected cells but not their mock counterparts, indicating that peroxisome uptake and the breakdown of very long chain fatty acids is required for the survival of endothelial cells latently infected with KSHV. While there does not appear to be an increase in the mitochondrial beta-oxidation of long chain fatty acids, very long chain fatty acid oxidation appears important during KSHV latency.

The role of fatty acid synthesis in EBV lytic and latent infection is less well understood. Fatty acid synthesis was shown to be important for EBV lytic replication [77]. The immediate early protein BRLF1 or R was shown to induce FASN in epithelial cells. A FASN inhibitor, C75, also inhibits the lytic reactivation of EBV induced by the R protein; however, not by another EBV gene, Z, that can also induce lytic replication. These data indicate that in epithelial cells, fatty acid synthesis is required for EBV at a distinct stage of lytic replication from KSHV. During EBV latent infection in nasopharyngeal carcinoma (NPC) cells—cells of epithelial origin—there is increased FASN expression as compared to uninfected NPC cells [78]. The EBV latent membrane protein 1 (LMP-1) is expressed in many but not all types of EBV-1 latency. LMP-1 can induce SREBP1 and FASN leading to increased lipid droplets [78]. The inhibition of SREBP1 signaling led to the decreased growth of LMP-1 positive cells in vitro and in vivo. Knockdown of LMP-1 also led to decreases in FASN in NPC cells, indicating the importance of LMP-1 in the induction of FASN. More work needs to be done to determine the importance of fatty acid synthesis in the different types of EBV latency and the different EBV-associated diseases.

### 4.4. Murine Herpesvirus 68

While elegant in vitro systems have been developed for the study of EBV and KSHV, the exquisite species specificity and ubiquitous nature of gammaherpesviruses hinders in vivo studies of these human viruses. The most well-established mouse model for the study of gammaherpesvirus biology is murine herpesvirus 68 (MHV68), an endemic pathogen of rodents that exhibits significant homology to the human gammaherpesviruses [79,80]. MHV68 readily infects laboratory mice, allowing for the utilization of powerful genetic and immunologic tools. Using the MHV68 model system, our group discovered a unique pro-viral role of the cellular mevalonate pathway [81]. The attenuation of cholesterol synthesis with statins significantly limits MHV68 replication in primary macrophages. Interestingly, through a series of rescue experiments in which intermediates of the mevalonate pathway were replenished in conjunction with statin treatment, we discovered that isoprenoids, rather than cholesterol itself, were responsible for facilitating viral replication. Statin treatment of intraperitoneally-infected wild-type mice resulted in a dramatic decrease in viral latency, but only a modest decrease in viral latency in mice that were infected intranasally. While the explanation for this route of infection-dependent phenomenon remains unclear, it is possible that the infected cell types or the level of interferon signaling may modify the antiviral activity of statins in vivo. In support of the latter hypothesis, statin treatment of intranasally-infected interferon alpha/beta receptor (IFNAR)-deficient animals profoundly reduced viral replication as well as subsequent viral latency. Thus, type-I interferon signaling modifies the interaction of MHV68 with the cholesterol synthesis pathway.

Like the other gammaherpesviruses, MHV68 likely usurps multiple metabolic pathways to facilitate viral replication and latency. Similar to that observed for human gammaherpesviruses, we found that fatty acid synthesis supports MHV68 replication, as evidenced by decreased viral replication following treatment with an inhibitor of acetyl-CoA carboxylase [82]. As cholesterol and fatty acid homeostasis are transcriptionally regulated by liver X receptors (LXRs), we set out to determine the extent to which LXR expression promotes MHV68 replication. Surprisingly, LXRs limited MHV68 replication in primary macrophages in vitro. Consistent with this antiviral function, LXR expression was elevated following the infection of primary macrophages in a type-I interferon-dependent manner. Further, this increase in LXR expression appeared to be a host response to limit, rather than induce, the expression of lipogenic LXR-target genes. The functional consequence of this transcriptional regulation is the repression of host lipid synthetic pathways, as LXR^-/-^ macrophages exhibit significantly greater rates of proviral cholesterol, fatty acid, and triglyceride synthesis.

In addition to the apparently redundant roles of LXRα and LXRβ to restrict lipogenesis in primary macrophages, LXRα exhibits unique antiviral properties during chronic MHV68 infection. We recently showed that LXRα^−/−^ mice exhibit peritoneal hyper-reactivation of MHV68 during long-term infection [83]. Importantly, no derepression of lipogenic genes was observed in the absence of LXRα, suggesting that increased fatty acid and cholesterol synthesis was not responsible for the observed increase in viral reactivation. Further, while LXRs have been purported to modulate inflammatory signaling in immune cells, we found no defect in the ability of LXRα-deficient animals to generate functional anti-MHV68 T cell responses; further, the antiviral capacity of interferons remained intact in the absence of LXRα. Interestingly, the expression of LXRα was important to support latent infection of peritoneal B cells, as MHV68 tropism was shifted towards peritoneal macrophages in LXRα deficient mice. Thus, LXRs, in addition to their classic role as master regulators of lipid synthesis, also exhibit unique characteristics that contribute to the control of chronic gammaherpesvirus infection.

### 4.5. Other DNA Viruses

While most of the research regarding lipid metabolism and DNA virus infection has been performed within the herpesvirus arena, there are a number of reports of other DNA viruses usurping, or otherwise manipulating, host lipid metabolism. In a study comparing the metabolic phenotypes of two cell lines commonly used for the production of adenovirus vectors, Silva et al. report substantial increases in glucose consumption and lactate secretion during the first 24 h post infection with Ad5 [84]. Additionally, infected cells exhibited increased choline consumption, suggesting an increase in phospholipid synthesis [84]. Changes in the utilization and production of other metabolites varied between cell lines and infection parameters. Human papillomavirus E5 protein has also been reported to modify cellular lipid pathways, with the most pronounced changes occurring in cholesteryl ester and glycerophospholipid synthesis [85].

With the exception of herpesviruses, poxviruses represent perhaps the best studied DNA virus family in the context of lipid metabolism. An early study of vaccinia virus (VACV) discovered that multiple viral proteins are modified by the cellular process of fatty acid acylation [86]. Specifically, the proteins M25 and M35 were identified to be both myristoylated and palmitoylated following a brief pulse with tritiated fatty acids. Interestingly, more robust palmitoylation occurred following a pulse with [^3^H]myristic acid than with [^3^H]palmitic acid, suggesting an inability of these particular viral proteins to be directly palmitoylated by pre-existing palmitic acid [86]. This group and others have followed up on these findings, characterizing the fatty acid acylation of many other VACV proteins [87,88,89,90,91,92,93,94]. In general, palmitoylation and myristoylation of VACV proteins facilitate viral capsid envelopment and egress from the infected cell [90]. The well-characterized VACV protein p37, for example, localizes to the trans-Golgi network and is required for proper virion envelopment [95,96,97]. Grosenbach and Hruby have demonstrated that the palmitoylation of p37 is required for this activity, and that a mutant VACV that expresses a non-palmitoylated p37 is virtually indistinguishable from a virus lacking p37 altogether [98].

VACV, like many of the herpesviruses, also utilizes cholesterol-laden lipid rafts during entry. Cholesterol depletion of BSC40 via methyl-β-cyclodextrin treatment profoundly reduced VACV infection [99]. Orynbayeva et al. utilized a novel approach in which the chromatic lipid-like polymer, polydiacetylene, was incorporated into vesicles and epithelial cells to examine virus–lipid interactions. Their report confirms the importance of the cell membrane lipid composition, as, of the different lipid compositions tested, VACV displayed the greatest affinity for cholesterol and sphingomyelin-containing lipid rafts [100]. Furthermore, Chung et al. discovered that multiple VACV proteins associate with lipid rafts [99]. Moreover, the lipid raft-associated protein, integrin β1, was reported to facilitate the cholesterol-dependent entry of VACV [101]. The current proposed role of integrin β1 is not only aiding in the attachment of the virus to the target cell, but also the activation of PI3K/Akt signaling that promotes endocytosis [101]. The cellular protein CD98 is also purported to be recruited to lipid rafts to facilitate integrin-dependent signaling required for VACV endocytosis [99]. Importantly, because VACV exists in multiple infectious forms and exhibits relatively broad cell tropism, there are likely multiple context-dependent entry mechanisms employed by the virus [102,103,104].

Following entry, VACV has been demonstrated to further manipulate cellular lipids by increasing de novo fatty acid synthesis [105]. Greseth and Traktman reported that the excess palmitate produced by the increased fatty acid synthesis facilitated viral replication not via palmitoylation or phospholipid synthesis, but rather as a means of ATP production through β-oxidation in the mitochondria [105]. Interestingly, VACV displays a particular reliance on β-oxidation and glutaminolysis to feed the citric acid cycle. The inhibition of fatty acid synthesis or β-oxidation had little effect on viral DNA and protein synthesis, but significantly abrogated virion assembly [105].

## 5. 25-Hydroxycholesterol

Lipids provide a backbone for the synthesis of numerous lipid-derived biosynthetic compounds and second messengers, including but not limited to prostaglandins, phosphoinositides, steroids, and diacylglycerol. While all of these compounds affect virus–host interactions, these effects are pleiotropic, tissue-specific, and further complicated by the ability of lipid derivatives to modulate immune responses. Thus, the complex regulation of virus–host interactions by fatty acid and cholesterol derivatives warrants an independent and comprehensive review. In contrast, in this review, we focus on 25-hydroxycholesterol (25HC), a cholesterol derivative with a broad and uniform antiviral activity. 25HC is an oxidation product of cholesterol synthesized by cholesterol 25-hydroxylase (CH25H) [106]. The expression of CH25H is stimulated by interferon signaling and interferon regulatory factor 1 [107,108,109], suggesting a role for 25HC in the response to viral infection. Indeed, it is now widely appreciated that 25HC exerts broad and potent antiviral functions [108,110]. 25HC restricts the replication of numerous RNA viruses, including hepatitis C virus [111,112,113], Zika virus [114], Lassa virus [115], reovirus [116], influenza virus [108], human rotavirus [117], human rhinovirus [117], and several others [110,118,119]. Additionally, 25HC restricts replication of human papillomavirus [117] and multiple herpesviruses [74,82,107,108,110,120,121]. In spite of the broad antiviral activity of 25HC, the mechanisms underlying such activity are still being elucidated.

One of the first described functions of 25HC was the attenuation of cholesterol synthesis [122,123]. Currently, 25HC is believed to limit cholesterol synthesis through two distinct mechanisms. Like cholesterol, 25HC prevents activation of SREBP2, the primary transcriptional regulator of cholesterol synthesis [124]. In fact, 25HC is a considerably more potent repressor of SREBP2 activation than cholesterol [124]. Interestingly, while repression of SREBP2 processing by cholesterol is believed to involve direct binding of cholesterol to SCAP [124], 25HC-mediated repression occurs via interactions between 25HC and Insig [125]. In addition to this transcriptional regulation of cholesterol synthesis, 25HC has been shown to promote the proteasomal degradation of HMGCR [126,127,128,129,130], the rate limiting enzyme of the mevalonate pathway. Thus, the end result of these two functions is the robust attenuation of cholesterol synthesis and, subsequently, viral replication. In support of this connection, Blanc et al. demonstrated that the antiviral activity of 25HC is significantly more pronounced under conditions where SREBP2 processing would be active [108]. Further, other oxysterols known to inhibit SREBP2 processing, specifically 27-hydroxycholesterol and 24(S), 25-epoxycholesterol, restricted replication of MCMV; in contrast, oxysterols that do not affect SREBP2 activation had no antiviral activity [108].

Additional antiviral mechanisms of 25HC are likely to exist. Liu et al. reported that the pretreatment of cells with 25HC significantly reduced the entry of multiple enveloped viruses by directly modifying cell membranes to attenuate viral protein-dependent fusion processes [110]. However, cholesterol synthesis and membrane cholesterol content were not examined in this study. Thus, while 25HC may modify the makeup of cellular membranes, it is not clear whether this a direct effect of 25HC, or an indirect effect due to global changes in cholesterol synthesis.

Similar to select other oxysterols, 25HC is an LXR agonist [131,132], although the extent to which this occurs in vivo is currently a topic of debate [133,134]. Nevertheless, Liu et al. have proposed that the ability of 25HC to activate LXRs contributes to its antiviral activity [135]. Interestingly, our group has reported that 25HC retains its antiviral activity against MHV68 in the absence of LXR expression [82]. Thus, considering these conflicting results, and the fact that 25HC is a relatively weak activator of LXRs [131,132,134], the interplay between the antiviral functions 25HC and LXRs remains to be further defined.

Moreover, several recent studies have reported an ability of 25HC to modulate inflammatory signaling pathways. Reboldi et al. [136] found that 25HC suppressed IL-1β expression as well as downstream IL-1-associated inflammation. Interestingly, Insig overexpression rescued IL-1β expression in CH25H^-/-^ macrophages, suggesting that the ability of 25HC to antagonize SREBP2 processing was involved in the suppression of IL-1β [136]. Accordingly, cells in which SCAP had been deleted by Cre-recombinase activity exhibited a diminished ability to express IL-1β in response to LPS [136]. Interestingly, Gold et al. found that 25HC exerts the opposite effect on many other inflammatory mediators, including IL-6, CSF1, and NOS2 [137]. Specifically, this group reported that 25HC potentiates the induction of these genes downstream of TLR activation, through a mechanism that is independent of LXR expression. They attributed this phenomenon to a 25HC-mediated increase in the expression of the AP-1 constituents, FOS and JUN, which correlates with increased AP-1 occupancy at CSF1 and IL-6 promoters [137]. Finally, they present an interesting observation in which expression of CH25H appears to be detrimental. They found that CH25H^-/-^ mice displayed significantly better survival and reduced pathology relative to wild-type control mice following a challenge with multiple influenza strains [137]. These findings correlated with reduced inflammatory gene expression in the lungs of CH25H^-/-^ mice, suggesting that the ability of 25HC to potentiate TLR-mediated inflammatory signaling may indeed have pathological consequences in the context of infection.

## 6. Concluding Remarks

Diverse viruses co-opt cellular metabolic pathways to facilitate virus infection. DNA viruses, most notably herpesviruses, are capable of inducing profound changes to cellular lipid synthesis. The resulting lipids have thus far been demonstrated to support viral entry, envelopment, egress, and signaling (e.g., palmitoylation and prenylation). Perhaps the most conserved use of lipids between viruses is the requirement for cholesterol-laden lipid rafts during viral entry. In some instances, specific lipids appear to be critical to viral replication. For instance, HCMV infection induces a marked increase in 26-carbon fatty acids which are incorporated into the viral membrane, and VACV encodes numerous proteins whose palmitoylation is required for efficient replication. While this review emphasized the critical roles of lipids during herpesvirus infection, it is likely that the importance of lipids in the context of other DNA virus infections will be unveiled in the coming years. Further, viruses routinely exploit host transcription factors to regulate viral gene expression. Interestingly, the ability of DNA viruses to usurp lipid-regulating transcription factors (such as SREBPs and LXRs) to directly regulate viral gene expression has not been reported and remains a curious possibility to be explored in future studies. Importantly, our understanding of the ways in which viruses hijack host lipids is currently superficial at best, and many of the mechanistic processes that underlie the viral manipulation of cellular lipid metabolism remain unknown. A better understanding of these mechanisms may offer new targets for antiviral therapeutics and provide opportunities to repurpose the numerous FDA-approved compounds targeting lipid metabolic pathways as antiviral agents.
